# Diagnosis and treatment of latent tuberculosis infection among household contacts in inland Bahia, Brazil: a cross-sectional follow-up study

**DOI:** 10.1590/1516-3180.2023.0339.R2.03072024

**Published:** 2024-12-20

**Authors:** Guilherme Lages Matias, Marcio Vinicius Ferreira Sales, Gabriela Santos Andrade, Brenda dos Santos Teixeira, Maria Eduarda da Macena Tenorio, Maria Augusta Vasconcelos Palácio, Maria Luisa de Carvalho Correia, Iukary Takenami

**Affiliations:** I Undergraduate Student, College of Medicine, Universidade Federal do Vale do São Francisco (UNIVASF), Paulo Afonso (BA), Brazil.; II Undergraduate Student, College of Medicine, Universidade Federal do Vale do São Francisco (UNIVASF), Paulo Afonso (BA), Brazil.; III Undergraduate Student, College of Medicine, Universidade Federal do Vale do São Francisco (UNIVASF), Paulo Afonso (BA), Brazil.; IV Undergraduate Student, College of Medicine, Universidade Federal do Vale do São Francisco (UNIVASF), Paulo Afonso (BA), Brazil.; V Undergraduate Student, College of Medicine, Universidade Federal do Vale do São Francisco (UNIVASF), Paulo Afonso (BA), Brazil.; VI Adjunt Professor, College of Medicine, Universidade Federal do Vale do São Francisco (UNIVASF), Paulo Afonso (BA), Brazil.; VII Dermatology and Sanitary Pneumology Service (SEDERPAS), Municipal Health Department, Paulo Afonso (BA), Brazil.; VIII Adjunt Professor, College of Medicine, Universidade Federal do Vale do São Francisco (UNIVASF), Paulo Afonso (BA), Brazil.

**Keywords:** Latent tuberculosis, Mycobacterium tuberculosis, Prevalence, Diagnosis, Treatment outcome, Latent tuberculosis infection, Household contacts, Tuberculin skin test, Tuberculosis control, Epidemiology

## Abstract

**BACKGROUND:**

The diagnosis and treatment of latent tuberculosis infection (LTBI) are crucial for tuberculosis (TB) control. Household contacts (HHC) of patients with pulmonary TB are at a high risk of LTBI due to their close proximity to source cases.

**OBJECTIVE:**

To describe the diagnosis and treatment of LTBI among HHC.

**DESIGN AND SETTING:**

This cross-sectional follow-up study was conducted in the municipality of Paulo Afonso, northeastern Brazil, between 2013 and 2022.

**METHODS:**

We retrieved secondary data from the medical records of HHC who were followed up at a specialized referral center for TB. LTBI prevalence estimates were calculated and are presented with 95% confidence intervals (CIs).

**RESULTS:**

In total, 622 HHC were screened for LTBI, with 620 evaluated using the tuberculin skin test (TST). Of these, 40 (6.5%) did not return for TST reading. The overall prevalence of LTBI was 53.1% (95% CI: 49–57.1%), with a high prevalence among females and individuals aged 25–34 years. The overall LTBI treatment initiation rate was 26.1% (95%CI: 21.5–31.3%), and 64.2% (95%CI: 53.3–73.8%) of HHC who initiated treatment completed their course.

**CONCLUSION:**

This study revealed a high prevalence of LTBI among HHC, particularly among women and individuals aged 25–34 years, underscoring the ongoing TB transmission within the community. Only 26.1% of the diagnosed HHC initiated treatment, with approximately 64% completing their course. This highlights the challenges in managing LTBI and emphasizes the need for targeted screening and interventions for high-risk groups.

## INTRODUCTION

Tuberculosis (TB), a disease caused by *Mycobacterium tuberculosis*, primarily affects the lungs but can also manifest in extrapulmonary sites.^1^ In 2019, TB impacted 10 million people worldwide, resulting in 1.5 million deaths and 85,338 new cases in Brazil. The emergence of coronavirus disease 2019 (COVID-19) has further complicated TB control, as reflected in Brazil’s 2021 statistics showing 78,833 new cases and a 16% increase in deaths.^2, 3^

In Brazil, the Northeast region, including the state of Bahia, faces a high TB burden, primarily affecting vulnerable populations in areas with limited healthcare access.^4^ Notably, the municipality of Paulo Afonso, situated 480 km from the capital of Bahia, had a TB incidence rate of 33 cases per 100,000 inhabitants in 2021, surpassing the state average.^5^ The challenge of disease control, particularly given the COVID-19 pandemic, which has exacerbated the TB epidemiological situation, requires a comprehensive plan of action across various fronts. This includes increased efforts to screen, diagnose, and treat latent TB infections (LTBI) as an integral component of public health interventions.

Approximately one-quarter of the world’s population is infected with *M. tuberculosis*. In general, these individuals remain asymptomatic, and approximately 5–10% develop the active form of the disease, with a higher risk of illness within the first 2 years after the initial infection. Although individuals with LTBI do not transmit *M. tuberculosis*, they remain reservoirs of the bacteria, which can be reactivated when immune system competence is compromised. Reactivation of LTBI is responsible for a large proportion of active TB cases, contributing to the perpetuation and maintenance of the disease transmission chain.^6, 7^

Therefore, diagnosing and treating LTBI are pivotal for reducing and eliminating TB, particularly within high-risk groups, such as people living with HIV (PLHIV); those undergoing immunosuppressive therapy, including TNF-α inhibitors; homeless persons; prisoners; illicit-drug users; healthcare workers; immigrants from high-TB-burden countries; and household contacts (HHC) of patients with pulmonary TB.^8^ HHC of patients with TB are at a higher risk of TB infection and disease due to prolonged and close exposure to the source case.^9^ A recent study noted that contacts accounted for more than 57.2% of LTBI treatment notifications.^10^ Sagili et al.^11^ found that a significant proportion of HHC of patients with TB in low- and middle-income countries have LTBI, with estimates suggesting that more than 50% of these contacts are infected. In a cross-sectional study conducted in Brazil, 48% of contacts of patients with pulmonary TB tested positive on the tuberculin skin test (TST), thereby meeting the criteria for LTBI treatment.^12^ Furthermore, factors, such as recurrent exposure, malnutrition, and a compromised immune system are known to influence infection rates among these high-risk populations.^13^

Given the significance of infection in the development of active disease, and in alignment with the international ‘End TB Strategy’ and national elimination goals, Brazil implemented the LTBI online surveillance vigilance system in 2018 to record LTBI diagnoses and treatment, along with a ‘Protocol for Latent Tuberculosis Infection Surveillance’. These initiatives have established ambitious goals and actions that have facilitated the expansion of screening coverage, diagnostic practices, and the implementation of shortened treatment regimens for LTBI.^14, 15^ Although these changes represent significant advances in TB control, the prevalence of LTBI remains unknown in most Brazilian regions due to the scarcity of studies.

## OBJECTIVE

To describe the diagnosis and treatment of LTBI among HHC who were followed up at a specialized reference center for TB in the municipality of Paulo Afonso, northeastern Brazil. The study aimed to generate information that best represents the local context, thereby aiding decision-making by managers in the control of LTBI and, consequently, avoiding the development of active TB.

## METHODS

### Study design and setting

This was a cross-sectional follow-up study. Initially, a retrospective cross-sectional analysis was conducted by examining the medical records of all HHC who underwent screening between 2013 and 2022 to determine the prevalence of LTBI. Subsequently, HHC diagnosed with LTBI were followed up longitudinally to monitor treatment progress according to the prescribed regimen.

This study was conducted at the ‘Dermatology and Sanitary Pneumology Service’ (SEDERPAS), a reference center for TB located in the municipality of Paulo Afonso, northeastern Brazil. The health unit serves as a referral center for the municipality of Paulo Afonso and neighboring regions, providing specialized services for comprehensive care in the management of leprosy and TB. It also conducts screening, diagnosis, and treatment of LTBI and is responsible for administering all skin tests in the region.

Paulo Afonso is located in the north of the state of Bahia, an important region bordering the states of Pernambuco, Alagoas, and Sergipe. According to the Brazilian Institute of Geography and Statistics (IBGE), the municipality has an estimated population of 112,870 inhabitants distributed in an area of 1,545.19 km^2^. According to the Health Department of Bahia, Paulo Afonso boasts 88% primary healthcare coverage, with a Human Development Index of 0.674.^16^

### Study population

The study population comprised HHC of patients with pulmonary TB residing in the Paulo Afonso municipality or nearby regions. An HHC was defined as an individual who resided in the same dwelling unit or plot of land as the index patient and shared the same housekeeping arrangements.^17^

### Selection criteria and follow-up

As part of the routine investigation at the specialized referral center, all HHC identified during the TB case investigation were invited for a screening visit. LTBI screening was performed using the TST, which was conducted by trained staff in accordance with the recommendations of the Brazilian Ministry of Health. The TST was conducted by intradermal injection (Mantoux method) of two tuberculin units of a purified protein derivative per 0.1 mL. The transverse induration diameter was measured 48–72 hours later. A TST with a cut-off point ≥ 5 mm was considered a positive result in the absence of clinical and radiographic signs of TB.^18^ Additionally, a chest X-ray was conducted to exclude active TB, and the absence of signs and symptoms was confirmed among the HHC.

The HHC underwent TST between 2013 and 2022, and their treatment followed the therapeutic regimen outlined by the guidelines of the Brazilian Ministry of Health.^18^ All individuals with a positive TST result were encouraged to initiate LTBI treatment. However, the patient made the final decision on whether to undergo treatment. Three treatment regimens were considered in this study: i) 6-month daily isoniazid at a dose of 5–10 mg/kg, with a maximum of 300 mg and at least 180 doses; ii) 4-month daily rifampicin at a dose of 10 mg/kg, with a maximum of 600 mg and at least 120 doses; or iii) 3-month weekly rifapentine (900 mg/week) plus isoniazid (900 mg/week), with at least 12 doses.^18^ Individuals undergoing LTBI treatment were asked to return every 30 days for refills. HHC who did not return for a follow-up clinical visit or any drug supply refills were considered lost to follow-up.

### Data collection

All data were extracted from the medical records housed at SEDERPAS. To achieve this, variables of interest were collected by double data entry to ensure reliability and accuracy in data collection. The study included variables such as sex (male or female), age group (in years: 0–4; 5–9; 10–14; 15–24; 25–34; 35–44; 45–54; or 55+), TST results (positive, negative, or did not return for TST reading), LTBI treatment regimen (isoniazid, rifampicin, or rifapentine + isoniazid), and outcome (completed treatment or non-completed treatment). Incomplete or missing data were excluded from analyses.

### Statistical analysis

For statistical analysis, the data were tabulated in Microsoft Excel spreadsheets and analyzed using the statistical software SPSS (version 22.0; IBM Corporation, Armonk, United States) and GraphPad Prism (version 8.0; GraphPad Software, San Diego, United States). Categorical variables are presented as absolute values or relative frequencies and were compared using Fisher’s exact test or the Chi-square test. The estimated prevalence of LTBI, along with the 95% confidence interval (CI), was calculated, as were the treatment initiation and completion rates. Quantitative variables are presented as mean and standard deviation (SD) and were compared using either Student’s t-test or Pearson’s correlation coefficient. Differences were considered statistically significant at P values < 0.05.

### Ethical considerations

This study was approved by the Research Ethics Committee (CEP) of the University Center of Rio São Francisco (UniRios) under opinion no. 4,858,939, in compliance with Resolution no. 466/2012 of the National Health Council. The requirement for informed consent was waived because the present study relied solely on secondary data. Authorization was obtained from the Municipal Health Department.

## RESULTS

**Figure 1:**
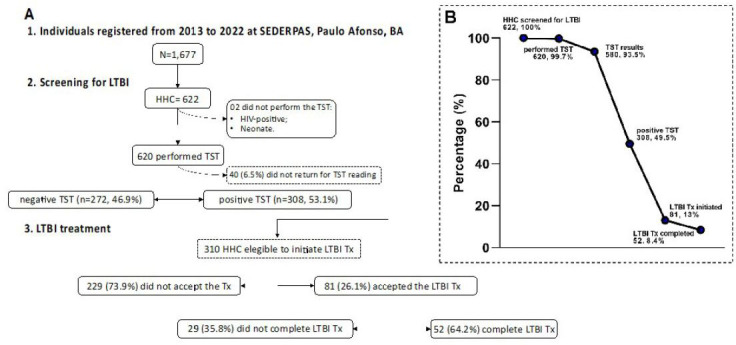
Flowchart illustrating the enrollment, latent tuberculosis infection diagnosis, and treatment of the study population (A). The chart shows the percentage of household contacts screened for latent tuberculosis infection, tuberculin skin test assessment, treatment initiation, and outcomes (B). Paulo Afonso, Brazil, 2013–2022.

A total of 1,677 individuals were included in this study. Among these, 622 (37.1%) were HHC of patients with pulmonary TB who underwent screening for LTBI. On average, approximately 62.2 ± 48.1 HHC were investigated per year. Two HHC did not undergo TST because they were HIV-positive and neonates. Of the 620 HHC who underwent TST, only 40 (6.5%) did not return for the reading (**Figure 1**). The demographic profiles of HHC who did and did not return for the TST reading were not significantly different (age, P = 0.72; sex, P = 0.42, data not shown).

HHC = household contacts; HIV = human immunodeficiency virus; LTBI = latent tuberculosis infection; TST = tuberculin skin test; Tx = treatment

**Figure 2:**
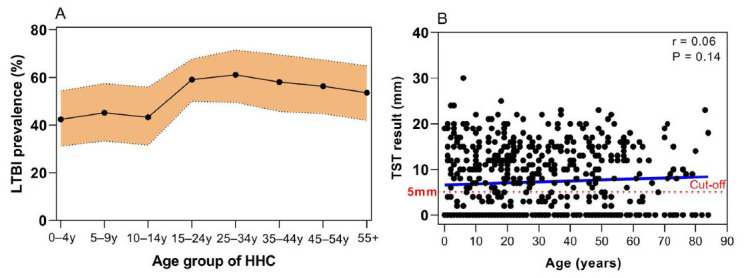
Prevalence rates of latent tuberculosis infection among household contacts by age groups and the correlation between tuberculin skin test results and the age of household contacts. Paulo Afonso, Brazil, 2013–2022.

Valid TST results were available for 580 (93.5%) of the 620 HHC. Using a cut-off of ≥ 5 mm of induration, 308 (53.1%) HHC had positive TST results. No significant differences in age were observed between the negative (26.7 ± 21.7 years) and positive TST groups (29.4 ± 20.2 years, P = 0.12), nor in sex distribution (negative TST, M/F = 112/160; positive TST, M/F = 122/186; P = 0.73). Similarly, no correlation was found between the TST results and the age of the HHC (r = 0.06, P = 0.14; **Figure 2B**).

The solid line represents the overall estimate of LTBI prevalence, and the dotted lines represent the 95% confidence interval by age group of HHC (A). The solid blue line depicts the linear correlation curve between the TST result and age of HHC, with a Pearson correlation coefficient of 0.06, P = 0.14. The red dotted line corresponds to the TST cutoff (B). HHC = household contacts; LTBI = latent tuberculosis infection; mm = millimeters; TST = tuberculin skin test.

The overall prevalence of LTBI among the HHC of patients with TB was 53.1% (95%CI: 49–57.1%). The prevalence according to sex and age group is shown in **Figure 2A** and **Table 1**.

**Table 1:** Prevalence of latent tuberculosis infection among household contacts by sex and age groups. Paulo Afonso, Brazil, 2013–2022

Characteristics	n (%)	LTBI	
		Prevalence	95%CI
**Sex**			
Male	122 (39.6)	52.1	45.8–58.4%
Female	186 (60.4)	53.8	48.5–58.9%
Age groups*			
0–4 years	28 (9.1)	42.4	31.2–54.4%
5–9 years	28 (9.1)	45.2	33.4–57.5%
10–14 years	26 (8.5)	43.3	31.6–55.9%
15–24 years	68 (22.1)	59.1	50.0–67.7%
25–34 years	44 (14.3)	61.1	49.6–71.5%
35–44 years	36 (11.7)	58.1	45.7–69.5%
45–54 years	40 (13.0)	56.3	44.8–67.2%
55+ Years	37 (12.1)	53.6	42.0–64.9%

*Data not available for one household contacts; LTBI = latent tuberculosis infection.

Among the 308 HHC with positive TST results, only 79 (25.6%) agreed to undergo LTBI treatment. Additionally, two HHC with treatment indications did not have TST results available. Thus, the overall LTBI treatment initiation rate was 26.1% (81/310; 95%CI: 21.5–31.3%), while the majority, 73.9% (229/310; 95%CI: 68.7–78.5%), declined treatment (**Figure 1**). No significant differences were found in terms of sex or age between individuals who consented to LTBI treatment and those who refused treatment. Interestingly, a slight difference was observed in the TST results between those who accepted treatment initiation and those who declined treatment (**Table 2**).

**Table 2:** Comparison of sex, age, and tuberculin skin test results between latent tuberculosis infection treatment acceptance and refusal among household contacts. Paulo Afonso, Brazil, 2013–2022

Variables	LTBI treatment, n (%)		P value
	Yes (n = 81)	Refused (n = 229)	
Sex			
Male	35 (43.2)	88 (38.4)	
Female	46 (56.8)	141 (61.6)	0.50
Age*			
0–4 years	6 (7.4)	23 (10.1)	
5–9 years	7 (8.6)	21 (9.2)	0.76
10–14 years	7 (8.6)	20 (8.8)	0.75
15–24 years	15 (18.5)	53 (23.2)	0.99
25–34 years	12 (14.8)	32 (14.0)	0.59
35–44 years	12 (14.8)	24 (10.5)	0.28
45–54 years	15 (18.5)	25 (11.0)	0.19
55+	7 (8.6)	30 (13.2)	0.99
TST (mm)			
Mean ± SD	14.4 ± 4.4	12.6 ± 4.7	0.003

*Age information was not available for one household contacts who declined LTBI treatment; LTBI = latent tuberculosis infection; mm = millimeters; SD = standard deviation; TST = tuberculin skin test.

Of the 81 HHC who initiated LTBI treatment, 59 (72.8%) received isoniazid monotherapy, seven (8.6%) received a rifampicin-containing regimen, and 15 (18.5%) received a combination of rifapentine and isoniazid. At the end of the follow-up period, 52 (64.2%) patients had completed treatment, and 29 (35.8%) were lost to follow-up (**Figure 1**, **Table 3**). The overall LTBI treatment completion rate was 64.2% (95%CI: 53.3–73.8%). Furthermore, no significant differences in sex, age, TST results, or treatment regimen were observed between individuals who abandoned or completed treatment (P > 0.005; **Table 3**).

**Table 3:** Latent tuberculosis infection treatment initiation among household contacts. Paulo Afonso, Brazil, 2013–2022

Variables	LTBI Treatment, n (%)		P value
	Complete (n = 52)	non-completion (n = 29)	
Sex			
Male	19 (36.5)	16 (55.2)	0.16
Female	33 (63.5)	13 (44.8)	
Age			
Mean (years) ± SD	29.4 ± 17.0	30.1 ± 20.0	0.87
0–4 years	2 (3.8)	4 (17.4)	0.55
5–9 years	5 (9.6)	2 (8.7)	
10–14 years	6 (11.5)	1 (4.3)	
15–24 years	9 (17.3)	4 (17.4)	
25–34 years	9 (17.3)	2 (8.7)	
35–44 years	9 (17.3)	3 (13.0)	
45–54 years	9 (17.3)	5 (21.7)	
55+	3 (5.8)	2 (8.7)	
TST (mm)			
Mean ± SD	14.8 ± 4.3	13.8 ± 4.6	0.32
Treatment regimen			
Isoniazid	37 (71.2)	22 (75.9)	0.69
Rifampicin	4 (7.7)	3 (10.3)	
Rifapentine + isoniazid	11 (21.2)	4 (13.8)	

LTBI = latent tuberculosis infection; mm = millimeters; SD = standard deviation; TST = tuberculin skin test.

## DISCUSSION

Studies providing knowledge and information about LTBI are crucial and necessary to achieve the goals of the WHO’s ‘End TB Strategy’ (2016–2035) and the Brazilian National Plan to End Tuberculosis proposed by the Brazilian National Ministry of Health. Therefore, this study was the first to estimate the prevalence of LTBI among HHC in the municipality of Paulo Afonso, Bahia, Brazil. This research is particularly important for HHC, who are at high risk of LTBI and progression to active TB.^8,9,19^

Our study identified an overall LTBI prevalence of 53.1% among HHC using a 5-mm TST cut-off. These results are consistent with those of a previous study conducted in Belém (Pará), where 52.3% of HHC who underwent TST yielded positive results.^20^ Conversely, other studies on LTBI prevalence in Brazil, conducted by Jones-López et al.^21^ and Fernandes et al.,^22^ reported higher prevalence rates of 62.4% and 66.4%, respectively. However, these studies used a ≥ 10 mm TST cut-off for their assessments. It is reasonable to assume that the differences in prevalence may be linked to the contagiousness of the index case. Although bacilloscopy data of the index case were not available in our study, Acuña-Villaorduña et al.^23^ investigated the relationship between HHC infection and the number of colony-forming units (CFU) of *M. tuberculosis* cultured in cough-generated aerosols from index TB patients in Vitória (Espírito Santo). They found that the frequency of TST ≥ 5 mm in contacts increased in a dose-response pattern with increasing aerosol CFU: aerosol negative (66%), aerosol low (69%), and aerosol high (79%).

In a meta-analysis that included studies from 36 countries, the overall population prevalence estimates were 24.8% (95%CI: 19.7–30%) and 21.2% (95%CI: 17.9–24.4%), based on the Interferon-Gamma Release Assay (IGRA) and a 10-mm TST cut-off, respectively.^24^ However, according to a systematic review and meta-analysis by Sagili et al.,^11^ the prevalence of LTBI among HHC of patients with TB was higher than that in the general population, estimated at 41% (95%CI: 33–49%). These findings underscore the need to prioritize LTBI screening for HHC, given that its prevalence in this group is higher than that in the general population.

The results based on sex and age group showed that the prevalence of LTBI is similar between men and women; however, some studies suggest that the prevalence may be slightly higher among men.^25^ On the other hand, a trend toward a higher LTBI prevalence was evident among individuals aged 25–34 years, regardless of sex. This age group includes individuals of reproductive and active working age, who are generally at a higher risk of *M. tuberculosis* exposure.^26^

The cut-off point for measuring skin induration in the TST was set at < 5 mm, with indurations of ≥ 5 mm indicative of *M. tuberculosis* infection. This measurement corresponds to the largest transverse diameter of the induration perpendicular to the forearm, and its correct reading is crucial because LTBI treatment is based on this cutoff point. TST results can be influenced by BCG vaccination or non-tuberculous mycobacteria (NTM) in the environment.^18^ However, the lack of BCG vaccination records among HHC in the present study hindered a more in-depth analysis. Nonetheless, despite the limitations of TST in identifying eligible individuals, a positive result likely indicates prior infection with *M. tuberculosis* and is not affected by BCG vaccination or NTM infection in endemic regions.^27^

Another limitation of the TST is the requirement for a second clinic visit after 48–72 hours to read the results. In such cases, a second test can be performed as soon as possible. According to our results, 6.5% of the participants did not return for the TST reading, most likely due to economic barriers, lack of time, non-adherence to the recommendations of health professionals, and poor knowledge about the importance of the TST.^28^ Similarly, Mendes et al.^20^ reported that only 2.3% of patients did not return for TST reading. Although a low frequency was observed, this does not reflect the reality in the municipalities of the country, in which the return rate ranges from 8.4% to 13.3%.^27,29, 30, 31^ These differences may be attributed to health professionals adopting a patient-centered approach and providing sufficient guidance during the test, which increases the quality of care.^19^

In the present study, no significant differences in sex or age were observed between individuals with negative and positive TST results. These findings are consistent with those of a study conducted at the Maruípe Health Unit in Vitória (Espírito Santo), where no significant differences were observed in sex and age between TST reactors and non-reactors.^32^ Similar results were also reported by Rogerio et al.^33^ These results suggest that sex and age did not significantly influence the TST outcome.

Treatment for LTBI has been recommended in Brazil since 1995 and is currently included in pillar 1 of LTBI surveillance as a crucial action to achieve its goals. However, most HHC in our study did not initiate LTBI treatment (73.9%) despite it being offered free of charge by the Brazilian Unified Health System.^15^ Unfortunately, the LTBI treatment initiation rate has been suboptimal in most studies, ranging from 33.9% to 87.8%.^12, 34^

Treatment was successfully completed by 64.2% of the HHC. This rate aligns with an estimated pooled rate of 65% (95% CI: 54–74%) reported in a systematic review and meta-analysis conducted by Sagili et al.^11^ Nevertheless, studies conducted in Brazil indicated a more favorable outcome, with success rates ranging from 53% to 83%.^12, 34^ No characteristic was found to be significant among those who completed treatment. However, according to Araújo et al.,^30^ a higher prevalence was observed among women.

Sociodemographic factors, including high transportation costs and limited social support, may have impacted treatment completion. Furthermore, factors such as medication palatability and extended treatment duration have been linked to a decreased likelihood of treatment completion.^11^ Moreover, inadequate health education, exemplified by recurrent communication gaps between healthcare professionals and individuals with LTBI, may also contribute to this issue.^35^

Despite the use of isoniazid and rifapentine as alternatives to shorten the treatment duration and mitigate the risk of treatment abandonment, the present study did not find any significant difference. This treatment regimen is recommended by the guidelines because of its effectiveness and short duration. However, further research is necessary to investigate local factors that may influence treatment outcomes and adherence among HHC.

This study has two primary limitations. First, the study relied on secondary data, which may have limitations in terms of accuracy and completeness. However, this study analyzed all reported cases of LTBI among HHC in the municipality, which enhanced the validity of the results. Second, the study analyzed a limited number of variables. Other critical factors that may influence treatment initiation and completion rates, such as socioeconomic status and educational level, were not investigated. A more comprehensive exploration of these modifiable factors is essential to develop effective interventions aimed at improving treatment uptake and adherence in at-risk populations. Additionally, IGRA, a more recent technology for diagnosing and assessing the prevalence of LTBI, was not employed in this study. This omission occurred because the Brazilian Ministry of Health continues to recognize TST as the preferred marker of infection in Brazil.

## CONCLUSION

This study provides important insights into the prevalence of LTBI and treatment uptake among HHC. The observation of an increased LTBI prevalence among women aged 25–34 years recently exposed to infectious TB in their households underscores the need for targeted interventions to address the needs of this group. Additionally, the low rates of LTBI treatment initiation and completion emphasize the urgency of identifying the reasons for these barriers and developing effective strategies to overcome them. Thus, to achieve TB control in municipalities, treating all individuals diagnosed with LTBI is crucial, as this can reduce TB transmission and prevent the development of active TB in high-risk populations.

Future research should investigate strategies to address barriers to LTBI treatment initiation and completion. Evaluating the effectiveness of targeted interventions and their impact on treatment outcomes will be essential for refining TB control strategies and enhancing public health efforts.

## References

[1] Moule MG, Cirillo JD (2020). *Mycobacterium tuberculosis* dissemination plays a critical role in pathogenesis. Front Cell Infect Microbiol.

[2] World Health Organization (2020). WHO consolidated guidelines on tuberculosis. Module 1: prevention – tuberculosis preventive treatment.

[3] World Health Organization (2022). Global tuberculosis report 2022.

[4] Cortez AO, de Melo AC, de Neves LO, Resende KA, Camargos P (2021). Tuberculosis in Brazil: one country, multiple realities. J bras pneumol.

[5] Brasil. Ministério da Saúde (2024). Departamento de Informática do Sistema Único de Saúde.

[6] Boom WH, Schaible UE, Achkar JM (2021). The knowns and unknowns of latent *Mycobacterium tuberculosis* infection. J Clin Invest.

[7] Qiu B, Wu Z, Tao B, et al (2022). Risk factors for types of recurrent tuberculosis (reactivation versus reinfection): a global systematic review and meta-analysis. Int J Infect Dis.

[8] Agbota G, Bonnet M, Lienhardt C (2023). Management of tuberculosis infection: current situation, recent developments and operational challenges. Pathogens.

[9] Velayutham B, Jayabal L, Watson B, et al (2020). Tuberculosis screening in household contacts of pulmonary tuberculosis patients in an urban setting. PLoS One.

[10] Pavinati G, Lima LV, Couto RM (2023). Indicação do tratamento da tuberculose latente: desafios identificados no sistema de notificação brasileiro, 2018-2022. Rev Saúde Pública Paraná.

[11] Sagili KD, Muniyandi M, Shringarpure K, et al (2022). Strategies to detect and manage latent tuberculosis infection among household contacts of pulmonary TB patients in high TB burden countries - a systematic review and meta-analysis. Trop Med Int Health.

[12] Wysocki AD, Villa TCS, Arakawa T, et al (2016). Latent tuberculosis infection diagnostic and treatment cascade among contacts in primary health care in a city of Sao Paulo state, Brazil: cross-sectional study. PloS One.

[13] VanValkenburg A, Kaipilyawar V, Sarkar S, et al (2022). Malnutrition leads to increased inflammation and expression of tuberculosis risk signatures in recently exposed household contacts of pulmonary tuberculosis. Front Immunol.

[14] World Health Organization (2017). The End Tb Strategy.

[15] Brasil. Ministério da Saúde (2022). Protocolo de vigilância da infecção latente pelo Mycobacterium tuberculosis no Brasil/Ministério da Saúde, Secretaria de Vigilância em Saúde, Departamento de Vigilância das Doenças Transmissíveis.

[16] IBGE (Instituto Brasileiro de Geografia e Estatística) População no último censo.

[17] Baliashvili D, Gandhi NR, Kim S, et al (2021). Resistance to *Mycobacterium tuberculosis* infection among household contacts: a multinational study. Clin Infect Dis.

[18] Brasil. Ministério da Saúde (2019). Manual de Recomendações para o Controle da Tuberculose no Brasil.

[19] Oxlade O, Benedetti A, Adjobimey M (2021). Effectiveness and cost-effectiveness of a health systems intervention for latent tuberculosis infection management (ACT4): a cluster-randomised trial. The Lancet Public Health.

[20] Mendes MJF, Rodrigues JP, Cruz da MS (2018). O rendimento da prova tuberculínica entre comunicantes de portadores de tuberculose pulmonar em Belém-PA. Enferm. foco (Brasília).

[21] Jones-López EC, Acuña-Villaorduña C, Fregona G, et al (2017). Incident Mycobacterium tuberculosis infection in household contacts of infectious tuberculosis patients in Brazil. BMC Infect Dis.

[22] Fernandes P, Ma Y, Gaeddert M, et al (2018). Sex and age differences in Mycobacterium tuberculosis infection in Brazil. Epidemiol Infect.

[23] Acuña-Villaorduña C, Schmidt-Castellani LG, Marques-Rodrigues P, et al (2018). Cough-aerosol cultures of Mycobacterium tuberculosis in the prediction of outcomes after exposure. A household contact study in Brazil. PLoS One.

[24] Cohen A, Mathiasen VD, Schön T, Wejse C (2019). The global prevalence of latent tuberculosis: a systematic review and meta-analysis. Eur Respir J.

[25] Lee SJ, Lee SH, Kim YE, et al (2014). Risk factors for latent tuberculosis infection in close contacts of active tuberculosis patients in South Korea: a prospective cohort study. BMC Infect Dis.

[26] Dolla CK, Padmapriyadarsini C, Thiruvengadam K, et al (2019). Age-specific prevalence of TB infection among household contacts of pulmonary TB: Is it time for TB preventive therapy?. Trans R Soc Trop Med Hyg.

[27] Machado A, Emodi K, Takenami I (2009). Analysis of discordance between the tuberculin skin test and the interferon-gamma release assay. Int J Tuberc Lung Dis.

[28] Monteiro ATA, Guariente de MHDM, Costa da AANF (2015). Prova tuberculínica: o controle da tuberculose em pacientes infectados pelo vírus da imunodeficiência humana (HIV). Semin Cienc Biol Saude.

[29] Silva APB, Hill P, Belo MTCT, et al (2016). Non-completion of latent tuberculous infection treatment among children in Rio de Janeiro state, Brazil. Int J Tuberc Lung Dis.

[30] Araújo NCN, Cruz CMS, Arriaga MB (2020). Determinants of losses in the latent tuberculosis cascade of care in Brazil: A retrospective cohort study. Int J Infect Dis.

[31] Picone CM, Freitas AC, Gutierrez EB, et al (2020). Access and adherence to isoniazid preventive therapy and occurrence of active TB in a cohort of people living with HIV: a retrospective cohort study in Sao Paulo, Brazil. Rev Inst Med Trop Sao Paulo.

[32] Lacerda TC, Souza de FM, Prado do TN (2017). Tuberculosis infection among primary health care workers. J Bras Pneumol.

[33] Rogerio WP, Prado do TN, Souza FM (2015). Prevalência e fatores associados à infecção pelo Mycobacterium tuberculosis entre agentes comunitários de saúde no Brasil, usando-se a prova tuberculínica. Cad Saúde Pública.

[34] Machado A, Finkmoore B, Emodi K, et al (2009). Risk factors for failure to complete a course of latent tuberculosis infection treatment in Salvador, Brazil. Int J Tuberc Lung Dis.

[35] Salazar-Austin N, Mulder C, Hoddinott G, et al (2022). Preventive Treatment for Household Contacts of Drug-Susceptible Tuberculosis Patients. Pathogens.

